# “Do We Know Jack” About JAK? A Closer Look at JAK/STAT Signaling Pathway

**DOI:** 10.3389/fonc.2018.00287

**Published:** 2018-07-31

**Authors:** Emira Bousoik, Hamidreza Montazeri Aliabadi

**Affiliations:** ^1^Department of Biomedical and Pharmaceutical Sciences, Center for Targeted Drug Delivery, School of Pharmacy, Chapman University, Irvine, CA, United States; ^2^School of Pharmacy, Omar Al-Mukhtar University, Dèrna, Libya

**Keywords:** janus tyrosine kinases (JAKs), signal transducers and activators of transcription (STATs), cancer, signaling pathways, proliferation, survival

## Abstract

Janus tyrosine kinase (JAK) family of proteins have been identified as crucial proteins in signal transduction initiated by a wide range of membrane receptors. Among the proteins in this family JAK2 has been associated with important downstream proteins, including signal transducers and activators of transcription (STATs), which in turn regulate the expression of a variety of proteins involved in induction or prevention of apoptosis. Therefore, the JAK/STAT signaling axis plays a major role in the proliferation and survival of different cancer cells, and may even be involved in resistance mechanisms against molecularly targeted drugs. Despite extensive research focused on the protein structure and mechanisms of activation of JAKs, and signal transduction through these proteins, their importance in cancer initiation and progression seem to be underestimated. This manuscript is an attempt to highlight the role of JAK proteins in cancer biology, the most recent developments in targeting JAKs, and the central role they play in intracellular cross-talks with other signaling cascades.

## Introduction

The fate of our cells is mainly decided by the intracellular signaling pathways that control mechanisms involved in phenotypical modifications. This crucial role becomes even more significant in cancer cells that rely upon a vast, complicated, and inter-connected network of signaling pathways for their survival and proliferation. Signaling pathways are mostly activated through cell membrane receptors that are triggered by different ligands, which initiate the mechanisms responsible for controlling phenotypical outcomes, e.g., proliferation, or apoptosis. For instance, Receptor tyrosine kinases (RTKs, including epidermal growth factor receptor or EGFR, and human epidermal growth factor receptor 2 or HER2) and cytokine receptors are among the most important cell surface receptors that activate these signaling cascades. It is well-established that cancer is a heterogeneous disease (1–5). In 2015, Sottoriva et al. proposed a “Big Bang” model of tumor initiation in colorectal cancer that suggests after initial oncogenic mutation, future generations acquire further mutations, which are present in discrete populations of cells that then expand as the tumor grows, leading to spatial heterogeneity (6). A similar and even more diverse pattern has been reported for other types of cancer. Amir et al. studied two human acute lymphoblastic leukemia samples with viSNE technology, and reported a large, irregular mass of abnormal cells that were more different than similar (7). The sub-population of a sample with intrinsic resistance to a therapeutic assault (due to different mutations in the target protein, and/or reliance on an alternative mechanism) would survive and outgrow other cells due to the selection pressure, and promotes relapse after therapy, which results in abundance of cells that were once minority (8). This “Darwinian clone selection” has been well-documented in different types of cancer cells in respond to a variety of molecularly targeted drugs (9). This inter- and intra-tumor heterogeneity means that each cancer cell potentially has access to a wide variety of mechanisms to arrive at the same phenotypical outcome.

In addition to the diversity of the signaling pathways, which provides ample opportunities for cancer cells to “switch” pathways as a response to molecularly targeted drugs, and to make the matters even more complicated, these pathways are also not completely independent, and are engaged in signaling cascade “cross-talk.” Recent findings have revealed extensive interactions between traditionally categorized cascades, which has blurred the line between parallel pathways. Many of the effector proteins seem to multi-task and be involved in different mechanisms. Activation of HER2, a tyrosine kinase membrane receptor specifically expressed in breast cancer cell membrane, triggers phosphorylation of RAF and Ras, which results in over-expression of Bcl-2 family proteins (10). Another widely inter-connected protein is MUC1, which is over-expressed in different types of carcinomas and is correlated with higher risk of metastasis and poor survival rate (10). MUC1 interacts with several cytoplasmic proteins and Ras-Raf-MEK-ERK signaling pathway (11), STAT3 (mediated by Src) (12), and proteins known to be activated by EGFR (13). Studies also indicate that crosstalk among signaling pathways contributes to a deregulation of PI3K–PTEN signaling that can lead to tumorigenesis (14). This could at least partially explain the growing number of preclinical data that indicate a failure to induce apoptosis despite effective inhibition of PI3K-Akt components (15, 16).

**Figure 1:**
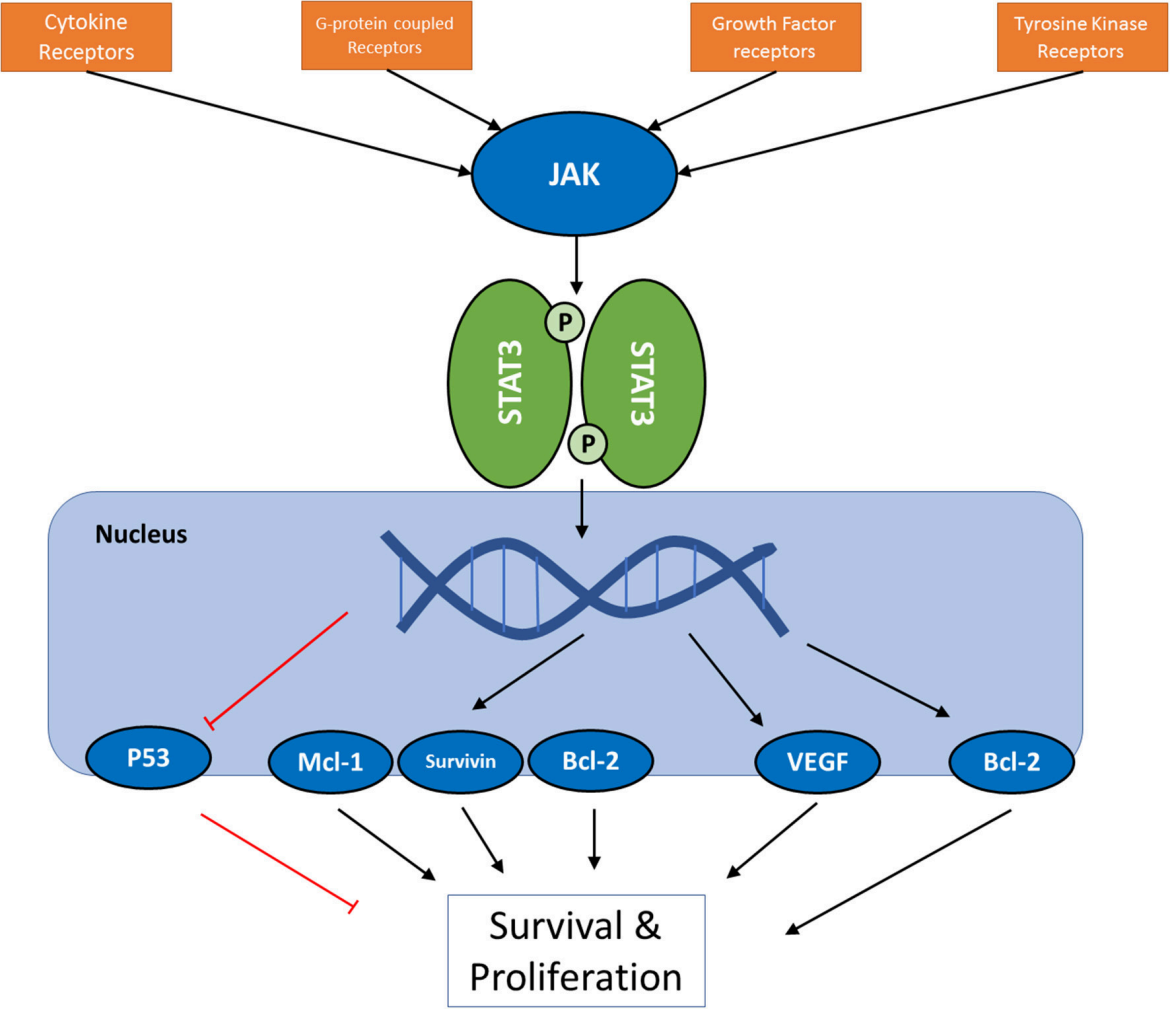
A schematic presentation of JAK/STAT pathway in cancer cells. For the full list of proteins regulated by the pathway via different STAT proteins, see Table 3.

Janus tyrosine kinase (JAK) and signal transducers and activators of transcription (STATs) are among the major proteins involved in this inter-pathway crosstalk, and latest reports have led to the elucidation of a key role of JAK/STAT signaling pathway in development, proliferation, differentiation, and survival of cancer cell, and in fact, Vogelstein et al. have included JAK/STAT pathway among 12 core cancer pathways (17). The effect of STAT3 activation on Ras and PI3K/Akt pathways (18), and the connections of JAK2 to PI3K and ERK pathways (19, 20) are examples of these inter-pathway cross-talks. JAKs are a family of proteins that belong to a category of intracellular non-receptor tyrosine kinases. In mammals, the JAK family contains four members: JAK1, JAK2, JAK3, and TYK2. STAT family is composed of seven members STAT1, STAT2, STAT3, STAT4, STAT5a, STAT5b, STAT6, which mainly act as transcription factors (21, 22). Compared to other major cell signaling pathways, JAK/STAT pathway seems relatively simple, with few components. Multiple review articles have focused on this important pathway and its role in cancer cells; however, this manuscript will try to take a closer look at the versatile mechanisms involved in this seemingly simple and straightworward cascade, and to analyze the efforts that have been made in altering its activity as a therapeutic strategy. Figure 1 summarizes the intracellular signaling cascades involving activation of JAK proteins.

## A brief history

JAK/STAT signaling cascade is among the highly conserved metazoan pathways observed in a wide range of species (23), which is involved in an array of cytokines and growth factors (22). The “story” of this silencing pathway has been previously reviewed in detail (24), where the authors track the origins of the discovery to 1950s and interferons. Our knowledge of existence of JAK/STAT pathway, however, is < 3 decades old. JAK1and JAK2 were identified in 1989, and the first member off JAK family of proteins was cloned and synthesized in 1990 (25). The gene was named Tyk2, and attracted attention due to a “kinase-like” domain next to the well-known and conserved protein tyrosine kinase domain (26). JAK name was taken from two-faced Roman God of doorways, Janus because the way that JAK possesses two near-identical phosphate-transferring domains: One domain exhibits the kinase activity while the other negatively regulates the kinase activity of the first (24, 27). Wilks et al. cloned and published the complementary DNA sequence for JAK2 in 1992, and demonstrated the same kinase/“pseudo-kinase” sections in the protein structure (28). In 1992 Shuai et al reported sequencing cDNA clones that were later called signal transducer and activator of transcription (STAT1; a and b) and STAT2 (29, 30). Later on, graduate students in Darnell lab cloned STAT3 and 4 from a lymphocyte cDNA library, establishing their membership in STATs family, and demonstrating that IL6 and EGF triggered phosphorylation of STAT3 (31, 32). The gene encoding STAT5 (initially named mammary gland factor; MGF) was cloned and sequenced in Groner's lab in 1994 (33). It was not until twenty first century that initial reports were published to link mutations in JAKs and STATs (resulting in persistent activation of the pathway) to several disorders (24).

## Mutations

Predictably, mutations that affect JAK/STAT pathway activity impact multiple cellular events (34, 35). The first report on mutation in this pathway surfaced in 1997, which explains the role of E695K mutation in the JH2 domain of JAK2 in murine cells that results in increased autophosphrylation and increased activation of STAT5 (36). First patient with confirmed mutation in the JAK/STAT pathway was reported in 2003 (37). Classical cases of growth hormone insensitivity (GHI) are usually due to mutations in GH receptor (GHR), which is a member of cytokine-hematopoietin family of receptors that do not show any intrinsic kinase activity (38). Dimerization of GHR leads to activation of JAK2, and in turn, activation of STATs. In 2003, a 16-years old Argentinian girl was diagnosed with severe growth retardation and immunodeficiency, which suggested involvement a pathway affecting both GH and cytokines (37). Immunoblotting showed absence of STAT5b (despite normal levels of total STAT5), and RT-PCR confirmed homozygous missense mutation in codon 630, resulting in substituting alanine with proline (39).

In 2005, four separate groups working on tyrosine kinase signal transduction reported a valine-to-phenylalanine mutation at position 617 in the JH2 domain of JAK2 that causes “gain-of-function” (40–43). To this day, V617F remains the most studied mutation in JAK family of proteins, and seems to be present in the majority of patient with Polycythemia Vera. This type of mutation is also frequently observed in other myeloproliferative neoplasms (44), and has been previously reviewed, extensively (45–48). Table 1 summarizes selected mutations (based on the significance and frequency of reports) in components of JAK/STAT signaling pathway. The list of all mutations reported in literature is out of the scope of this manuscript.

**Table 1:** Selected JAK/STAT mutations and resulting disorders.

**JAK/STATs**	**Mutation**	**Disease**	**References**
JAK1	G871E	Uterine leiomyosarcomas	(49)
	S703I	Inflammatory Adenoma and Leukemia	(50)
JAK2	V617F	Proliferative Neoplasms	(41)
	K539L	Polycythemia Vera	(51)
	T875N V625F	Myeloproliferative neoplasms	52 53
	JAK2Δ/REED	Acute lymphoblastic leukemia	(54)
JAK3	A572VV722IP132T	Acute megakaryoblastic myeloid leukemia	(55)
	M511I	Prolymphocytic leukemia	(56)
STAT1	L706S	Impairment of mycobacterial immunity	(57)
STAT3	Y640F	Large granular lymphocytic leukemia	(58)
	D661Y		
	D661V		
	N647I		
STAT5b	N642H	T-cell acute lymphoblastic leukemia	(59)
STAT6	P419D/G	Follicular Lymphoma	(60)

## Triggering the signal: receptors

JAK/STAT signaling begins with the activation of JAK by binding of a ligand such as growth factors, interferons, or interleukins to specific transmembrane receptors. A wide array of receptors has been associated with JAK/STAT pathway activation, which are summarized in Table 2. The cytokine receptors are probably the most commonly known family of transmembrane receptors associated with JAK activation. Generation of knockout mice for JAK family members, and evaluation of response to cytokine stimuli has contributed significantly to our understanding of the relationships between JAKs and cytokine receptors, which has led to the belief that cytokine receptors each prefer specific member(s) of JAK family for the signal transduction effector (109, 110). However, homozygous deletion of JAK1 and JAK2 causes lethality in mice due to disruption of neuronal development (111) and definitive hematopoiesis (110), respectively.

**Table 2:** A summary of the receptors involved in activating JAK/STAT pathway in cancer.

**Receptors**		Cancer type^*^	**Activated JAK**	**Activated STAT**	**References**
G protein-coupled receptors (GPCR)	5-HT2AR	Breast	JAK2	STAT3	(61)
	CCR 2	Squamous cell	JAK2	STAT3	(62)
	CCR 5	Breast	JAK1	STAT5	(62)
	CXCR4	GI; Breast	JAK2,3	STAT1,2,3,5	(63)
	PAFR	Breast; Hepatocellular	JAK2; TYK2	STAT1,2,3,5	(64), (65)
	PAR 1	Breast	JAK2	STAT1,3	(66)
	BDKRB2 (B2R)	Ovarian; Pancreatic	TYK2	STAT3	(67)
	AT1 R	breast	JAK2; TYK2	STAT1,2,3,5	(68), (69)
Cytokine receptors	IL-2 R	Glioma; Breast	JAK1,2,3	STAT1,3,4,5	(70), (71)
	IL-3 R	Hematologic	JAK1,2	STAT5	(72)
	IL-4 R	Cervical; Ovarian; Liver	JAK1,3	STAT6	(73)
	IL-5 R	Leukemia	JAK1,2	STAT1,5	(74), (75)
	IL-6 R	Breast	JAK1,2; TYK2	STAT1,3	(73), (76)
	IL-7 R	Multiple Types	JAK1,3	STAT1,3,5	(77), (78)
	IL-9 R	Ovarian; Pancreatic	JAK1,3	STAT1,3,5	(79), (80)
	IL-10 R (α & β)	Multiple Types	JAK1; TYK2	STAT1,3	(73), (81)
	IL-11 R	Breast; Prostate	JAK1,2	STAT1,3	(82)
	IL-12 R	Ovarian; Melanoma	JAK2; TYK2	STAT1,3,4,5	(83)
	IL-13 R	Multiple Types	JAK1,2; TYK2	STAT1,3,5,6	(84)
	IL-15 R	Colorectal	JAK1,3	STAT3,5	(76)
	IL-20 R	Multiple Types	JAK1	STAT1,3	(85)
	IL-21 R	Multiple Types	JAK1,3	STAT1,3,5	(86)
	IL-22 R	Colorectal	JAK1; TYK2	STAT3	(87)
	IL-23 R	Squamous cell carcinoma; Breast	JAK2; TYK2	STAT1,3,4,5	(83), (88)
	IL-24 R	Multiple Types	JAK1	STAT3	(89)
	IL-27 R	Multiple Types	JAK1,2; TYK2	STAT1,2,3,4,5	(90)
	IL-31 R	Lymphoma	JAK1,2	STAT1,3,5	(91)
	IFN α & β	Multiple Types	JAK1; TYK2	STAT1,2,3,4,5	(92)
	IFN γ	Multiple Types	JAK1,2	STAT1,3,5	(92)
	IFNλ (IL28/29)	Multiple Types	JAK1; TYK2	STAT1,2	(93)
	GM-CSFR	Melanoma	JAK2	STAT5	(94)
	G-CSFR	Cervical; Thyroid	JAK1,2	STAT1,3,5	(95)
	Leptin R	Breast	JAK2	STAT3	(96)
Receptor tyrosine kinases	EGFR	Multiple Types	JAK1,2	STAT1,3	(97)
	Insulin R	Multiple Types	JAK2	STAT1	(98)
	FGFR	Multiple Types	JAK2	STAT1,3	(99), (100)
	PDGFR	Glioma; Breast	JAK2	STAT1,3,5,6	(101), (102)
	VEGFR	All solid tumors	JAK2	STAT1,3,5	(103)
	TrkR	Breast Cancer	JAK2	STAT3	(104)
	TieR	Multiple types	–	STAT1	(105)
Homodimeric hormone receptors	EPOR	Breast	JAK2	STAT3,5	(71)
	PRLR	Breast	JAK2	STAT1,3,5	(106)
	GHR	Multiple Types	JAK2	STAT1,3,5	(107)
	TpoR	Myeloproliferative	JAK2; TYK2	STAT1,3,5	(108)

* *While the receptors included in the table are obviously expressed in multiple types of cancer, the specified type is related to the reference cited, and the link to the JAK/STAT pathway*.

Cytokine receptors activate JAK/STAT pathway through a variety of combinations of different JAK and STAT family members, which highlights the versatile nature of this pathway. The receptors in this family that are linked to JAK activation could be categorized as interleukin (IL) receptors, interferon (IFN) receptors, and colony stimulating factor receptors (CSFRs). Among IL receptors, gp130 subunit and receptors for IL-2, IL-3, IL-4, IL-6, IL-7, IL-9, IL-10, IL-11, IL-12, IL-13, IL-15, IL-20, IL-21, IL-22, IL-23, IL-27, IL-31, and Leptin have been reported to trigger activation of specific members of JAK family of proteins; however, while JAK1 seems to be a common factor among them (except for IL-12 and IL-23 receptors), a wide range of combinations of downstream effectors are observed. For example, heterodimerization of the IL-2Rβ and γc cytoplasmic domains has been reported to activate JAK1 and JAK3, with JAK1 associating with IL-2Rβ and JAK3 with γc (70). Interaction of IL-2 and the receptor, mostly results in activation of STAT5; however, STAT3 and STAT1 are also activated to lower degrees (112). Crucial role of IL-2 and its receptor in development of breast tumors, and a correlation between the malignancy of the tumor and expression of these receptors has been reported (113). The erythropoietin receptor (EPOR) is a hormone receptor that shares extra-cytoplasmic structural characteristics with cytokine receptor family [EPOR and IL-2Rβ share 45% amino acid identity in box 1 and box 2 cytoplasmic regions (114)]. In 1994, D'Andrea and Barber reported that EPOR stimulation results in rapid and dose-dependent JAK2 phosphorylation (71). On the other hand, simulation of IL-3 receptor via treatment with proteasome inhibitor, N-acetyl-L-leucinyl-L-leucinyl-norleucinal (LLnL) has resulted in prolonged activation of JAK 1 and 2, and stable phosphorylation of STAT5 (72).

It has been suggested that different cytokine receptors preferentially use one of the members of JAK family of proteins, or a specific combination (115). However, the mechanism of this selectivity is unclear. Interestingly, IL-4R (73) and IL-13R (84) are the only cytokine receptors that transduce the signal to STAT6. STAT6 has different functions in different cell types, and activates the transcription of a different set of proteins in T cells compared to non-lymphocyte cells (116). IL-13R is expressed in different tumor types, and although breast cancer cells are not among those, in a recent study Kawakami et al. reported targeting breast cancer cells by IL-13, after transfecting the cells with IL-13Rα2 plasmids (117). The receptor for IL-5 is a crucial factor in the physiology of eosinophils, multifunctional granulocytes that play a role in immune system, and are associated with the pathology of asthma and inflammation (118). STAT1 and STAT5 are activated through the signaling triggered by this receptor (Table 2).

IL-6 and IL-10 predominantly activate STAT3 with sometimes diverse outcomes (73, 76). Both STAT1 and STAT3 have been reported to be activated via IL-6R signal; however, different cell types show strong preference toward one STAT. SOCS3 is a protein that is induced via STAT signaling from different cytokine receptors, and acts as a feedback inhibitor on the expression of IL-6R (among other receptors). In the absence of SOCS3, STAT3 activation is significantly increased (119). However, STAT1 activation is not affected similarly, and therefore, in presence of SOCS3 the path activated by IL-6R switches from STAT3 to STAT1 to some extent (120). Even though IL-10R signaling resembles IL-6R pathway closely, the STAT3 activation of IL-10 induces transcription of a different set of proteins that are mostly involved in inhibition of inflammatory responses (121). IL-12R and IL-23R are structurally related, use the same signaling pathway, and are among the cytokine receptors that require TYK2 for their signal transduction (73). In T-cells, IL-12R activation results in STAT4 stimulation, which induces IFN-γ expression. IL-11 and its receptor have been indicated in breast cancer development and progression, and in 2006 IL-11 was reported as a predictor of poor prognosis in this type of cancer (122). IL-31, mainly produced by CD4(+) T cells and is a member of the gp130/IL-6 cytokine family. IL-31R activates JAK/STAT, PI3K/AKT, and MAPK pathways and acts on a broad range of cells (91). While other IL receptors [e.g., IL-19 (123) and IL-35 receptors (124)] have been reported to activate JAK/STAT pathway, their role in cancer cells is unclear.

TYK2 is the main difference between the pathways activated by type I (IFNα and β) and type II (IFNγ) IFN receptors. It has been reported that IFNαR1 and R2 (β) are associated with TYK2 and JAK2, respectively, while IFNγR1 and R2 activate JAK1 and JAK2 respectively (92). Briscoe *et al*. reported that JAK1 negative U4A cells demonstrate a partial response to IFNγ; however, the JAK2 negative γ2A cells did not response to IFNγ at all (125). IFNγ predominantly triggers activation of STAT1. There is evidence that IFN receptor activation triggers other intracellular proteins involved in other signaling pathways, including MAP kinase, PI3-K, CaMKII and NF-κB (126). Granulocyte Colony Stimulating Factor (G-CSF) and Granulocyte/Macrophage Colony Stimulating Factor (GM-CSF) are among other cytokines that have been linked to JAK/STAT pathway activation. G-CSFR is reported to mainly activate JAK2 and STAT3, and is expressed in several normal and malignant tissue (95). GM-CSF receptors have been identified on most types of myeloid progenitors, mature monocytes, neutrophils, eosinophils, basophils, and dendritic cells and mainly contribute to defense mechanisms against bacterial infections (127). GM-CSFR is also reported to activate JAK2; however, STAT5 is reported to be the main member of STAT family of protein to be activated via this pathway (94). Leptin is a cytokine normally secreted from adipose tissue, and is involved in regulating energy consumption and appetite (128). Interaction of leptin with the leptin receptor (which is categorized as a type I cytokine receptor) initiates the signaling cascade by phosphorylating associated JAK2. This, in turn activates STAT3, and MAPK extracellular signal-activated kinase 1/2 (ERK1/2) (128). Leptin signaling pathway has been reported to play a role in the proliferation of breast cancer cells via JAK/STAT, ERK1/2, PI3K-Akt pathways, and by enhancing angiogenesis through up-regulating vascular endothelial growth factor (VEGF) (96).

While JAK/STAT pathway was originally identified as a pathway activated by IFN signaling cascades, it has been recently reported that JAK proteins can be activated by other types of receptors to widen the array of the signals that could trigger this signaling pathway. G protein-coupled receptors (GPCR) are one of the categories of receptors that have shown capability to activate JAK (129). Among GPCRs, CXCR4 has been indicated to play a role in breast cancer cell growth. This receptor, activated by chemokine stromal cell-derived factor (SDF-1alpha), becomes tyrosine phosphorylated through activation and association with the receptor of JAK2 and JAK3 kinases, which in turn recruit and tyrosine phosphorylate multiple STAT family members (63). In this category of receptors, platelet-activating factor receptor (PAFR), bradykinin B2 receptor (B2R), and angiotensin II receptor type 1 (AT1R) all activate TYK2 (along with JAK2 for PAFR and AT1R) to trigger the JAK/STAT pathway. Among GPCRs, 5-HT2A receptor (5-HT2AR) has been identified as the main receptor to mediate the cell growth enhancing effect of serotonin (5-hydroxytryp-tamine; 5-HT) in different tissue, including MCF-7 breast cancer cells (130). The signal transduced through this membrane is known to activate both Ras/Raf and JAK/STAT pathways, and a recent study confirmed activation of JAK2/STAT3 combination by this receptor in JEG-3 human trophoblast choriocarcinoma cells (61). Receptors to both families of chemokines (CC and CXC) are also known to activate JAK/STAT signaling pathway. In a 2001 manuscript, Mellado et al. identified JAK1 (but not JAK2 or 3) to be associated with CCR5, while CCR2 promoted JAK2 activation in HEK-293 cells transfected with CCR5 and CCR2, respectively (62). On the other hand, CXCR4 has been found to be a prognostic marker in a variety of cancers, including breast cancer (131). Activation of CXCR4 receptor by chemokine stromal cell-derived factor (SDF-1α) has been reported to activate JAK2 and JAK3 independent of Gα_i_-1, and in turn recruit several members of STAT family (63). Angiotensin II also activates JAK/STAT pathway via AT1 receptor (AT1R) (68). In 2000, Ali et al. reported activation of JAK2/STAT1 combination that was independent of the tyrosine residues of the receptor, but completely dependent on the catalytic activity of JAK2 (69).

Activation of STAT family of proteins by RTKs have been long speculated; however, involvement of JAK proteins in the process has been a topic of debate. A possible link between signals transduced by epithelial growth factor receptor (EGFR) and STAT family activation has long been identified (132). However, the exact mechanism was not clear. In 2004, Andl et al. reported a JAK-dependent activation of STAT1 and 3, using a specific JAK inhibitor (AG-490), and suggested that EGFR induces the phosphorylation of STAT1, which triggers complex formation of STAT1 and 3 with JAK1 and 2 (97). Other reports since then have confirmed this link, and indicated the regulation of PD-L1 expression, among other intracellular roles, via this link (133–135). Among other growth factor receptors, fibroblast growth factor receptor (FGFR), platelet-derived growth factor receptor (PDGFR), and vascular endothelial growth factor receptor (VEGFR) have also been linked to this cascade. FGFR has been reported to stimulate STAT1 and 3 through JAK2 (among other downstream proteins) (100). It has been reported that tyrosine phosphorylation of STAT3 via this receptor is JAK-dependent, relying on formation of a complex by JAK2 and Src with FGFR1 (99). On the other hand, a recent study has indicated VEGFR-2 to activate the JAK2/STAT3 signaling axis by recruiting JAK2 and STAT3, which results in over-expression of MYC and SOX2 (136).

There are a few reports that claim toll-like receptors (TLRs) could also activate STAT3, which is one of the pathways for this family of receptors to play a role in tumor development (137); however, their involvement is contentious. TLR4 and TLR9 are among the receptors that have shown the most significant correlation with STAT3 activation. It has been reported that TLR4 overexpression could lead to STAT3 activation in intestinal epithelial cells, which also correlates with the clinical outcomes of colon adenocarcinoma (138). Upregulation of IL-6 by TLR4 is also reported in lymphoma (139), as a possible mechanism in the carcinogenesis. TLR9 is overexpressed in glioma stem cells, and a correlation between the expression level of TLR9 and survival rate in glioblastoma has been reported (140). It has been speculated that TLR9 activates JAK2 via Frizzled 4, which results in phosphorylation of STAT3 (141).

Hormone receptors are another family of receptors that have been associated with JAK/STAT pathway. In addition to EPOR, prolactin receptor (PRLR) has also been indicated in the activation of this signaling cascade. In 1997, Pezet et al. reported that binding of prolactin to its receptor results in dimerization of JAK2, which is “constitutively” associated with this receptor (142). It has been speculated that JAK/STAT is the principal signaling cascade activated by PRLR (106). Although considered an “archetypal” cytokine receptor, growth hormone receptor (GHR) is also associated with JAK2 activation and triggers JAK/STAT pathway (107).

## JAK activation

Unlike RTKs, cytokine receptors do not possess intrinsic kinase domain, and therefore, rely on JAK family to transfer the signal to the cytoplasmic components of the cascade (143). JAK is associated with cytoplasmic domains of cytokine receptors via JAK binding sites that are located close to the membrane and forms a complex that is equivalent in function to RTKs (144). However, the where and when of this association has been a topic of discussion (73). Members of JAK family consist of seven different JAK homology (JH) domains that include a four-point-one, ezrin, radixin, moesin (FERM) domain (JH5, 6, and 7) and a Src homology 2 (SH2) domain (JH3, and 4). JH1 and JH2 form the kinase and pseudokinase domains, respectively (145). The N-terminus half of all four members comprises FERM and SH2 domains that associate JAK with the cytoplasmic tail of the cytokine receptors (146). Binding of the ligand to cytokine receptor reorients the receptor/JAK dimers, which brings the JAKs close enough to transphosphorylate the partner JAK in the dimer at JH1. The activity of JH2 domain has only been reported for JAK2, as 10% catalytic activity compared to JH1 (133), and had been speculated to play an auto-inhibitory role, since the loss of JH2 leads to constant activity (147). Activated JAKs in turn, phosphorylate the residues on the cytoplasmic tail of the cytokine receptor to create “docking sites” for recruitment of downstream proteins with SH2 domains, e.g., STAT family of proteins (145). It is evident that different receptors have specific preferences for the JAK family protein they use as signaling effector, which means there is an obligate relationship between the receptor and the specific JAK protein(s) activated (143). However, in many cases, it has been shown that in the absence of the specific JAK family member, other proteins in the family have taken the responsibility and transferred the signal.

## Recruitement of stats and transport to nucleus

The next step in this signaling cascade is the recruitment of members of STAT family of proteins. Inactivated (or “latent”) STATs are found in cytoplasm [although, non-canonical mechanism of activation indicates presence of non-phosphorylated STATs in nucleus (148)]. As their name indicates, STATs act both as signal transducer and transcription factor; however, two structural components make them unique among transcription factors: an SH2 domain and a highly conserved C-terminal tyrosine residue (149). It is this tyrosine residue that is phosphorylated by activated JAKs. After phosphorylation, STATs form stable homodimers or heterodimers with other STAT proteins via SH2 domain interactions (150). A similar specificity observed with JAKs is seen here as well, where specific members of STAT family respond to a defined set of stimuli and receptors. Among STATs, STAT3 has been shown to be activated through other pathways, most importantly via EGFR and SRC (31, 151). The JAK/STAT activation could also be inactivated by negative regulators, e.g., SH2-containing protein tyrosine phosphatase (SHP) and suppressor of cytokine signaling (SOCS) proteins (152).

After activation by tyrosine phosphorylation, STATs become dimerized and translocate into the nucleus, where they act as transcription factors. Most STATs form homodimers; however, heterodimer formations (including STAT1/2, STAT1/3, and STAT5a/b) have also been reported (153). STAT1 has been reported to exist as pre-formed homodimers, and phosphorylation induces reorientation (anti-parallel to parallel conformation), which presumably could be true for other STATs as well (154). While translocation between cytoplasm and nucleus is a regular occurrence, the nuclear envelope provides a barrier that prevents free diffusion of large molecules (more than 40–60 kilo Dalton in molecular weight). These large molecules, including STATs usually require a specific transport receptor for facilitated transport (155). The receptors involved in importing molecules into nucleus are commonly known as importins, which consist of α and β subunits, known as importin α and β, respectively (156). The protein to be imported into the nucleus binds to the importin α via a specific motif on the protein called nuclear localization sequence (NLS) (157). After binding to the protein, importin α interacts with importin β, which docks the protein/importin complex at nuclear core complexes (NPCs). The translocation process is an active transport that requires energy, which is provided by NPC-associated GTPase, known as Ran (156). The translocation of STATs into nucleus via importins is a subject of discussion. For example, there are six human importin αs reported in literature, which show some similar structural characteristics, but binding specificity as well. While it seems accepted that STAT1 and STAT5 are transported into nucleus by importin α5 and α3, respectively, the same certainty does not seem to exist for STAT3. Different reports indicate involvement of importin α5 and α7 (158), importin α3 and α6 (limited to testis) (159), or a variety of importin αs (160). On the other hand, there are speculations that STATs do not contain functional NLS altogether, and therefore, NLS-containing chaperons are required to associate with STATs to facilitated binding to importins (153). Other reports indicate a binding site on STAT1 and STAT3 slightly different than the NLS binding site observed on other proteins (161). STAT3, 5, and 6 could be translocated into nucleus in the un-phosphorylated form as well (162).

## Stats as transcription factors

STATs have demonstrated the capability to activate the transcription of non-active genes in a few minutes (163). STAT family of proteins play multiple roles in cancer cells, and specifically, STAT3 has been shown to enhance cancer cell proliferation, migration, and survival, in addition to suppression of antitumor immune response (137). JAK activation is not the only mechanism of the activation of STATs and their migration into nucleus. For instance, a link between STAT activation and Src family of kinases has been proposed by researchers, which will promote the transcription of proteins such as VEGF and IL-8 (164). This ability of STATs to integrate the signal from a wide variety of signaling cascades indicates the possibility of regulation of a variety of genes through this family of transcription factors that serve different mechanisms involved in growth, differentiation, and survival. Among STATs, STAT1 and STAT2 are known as the targets of interferon activation (165). However, activation of STAT1 via PDGF has also been reported (166). STAT1 forms a heterodimer with phosphorylated STAT2, and IFN-regulatory factor 9, and is transported into nucleus to bind to IFN-stimulated response element (ISRE) in promoters of the responsive genes (167, 168). STAT1 homodimers are also formed. Both dimers seem to promote the expression of genes that enhance growth arrest and apoptosis (Table 3). For instance, STAT1 is involved in expression of several caspases, as executives of apoptosis (169). Based on the downregulation and activation pattern of downstream proteins, STAT1 seems to be involved in controlling the cell growth, enhancing vascularization, and inducing cell death, which are all characteristics that inhibit tumor growth.

**Table 3:** Selected survival-related genes regulated by members of STAT family of proteins.

**STAT**	**Downstream target**	**Change in expression**	**Function (Outcome)**	**References**
STAT1	Caspase 2,3,7	↑	Induces Apoptosis	(169)
	Fas	↑	Death receptor (Apoptosis)	(170)
	Fas-L	↑	Ligand for Fas (Apoptosis)	(171)
	TRAIL	↑	Ligand for TNF (Apoptosis)	(170)
	XAF1	↑	Antagonizes XIAP (Apoptosis)	(172)
	IRF1	↑	Transcription Factor (Apoptosis)	(173)
	P21 (CDKN1A)	↑	Inhibitor of cyclin D (cell cycle arrest)	(174)
	P27 (CDKN1B)	↑	Inhibitor of cyclin D (cell cycle arrest)	(175)
	Socs1/3	↑	Negative feedback/pro-inflammatory	(176)
	IL-12	↑	Negative feedback/pro-inflammatory	(175)
	IFITM1	↑	Antiproliferative	(177)
	CXCL10	↑	Angiogenesis (Tumor growth)	(178)
	Bcl-2	↓	Anti-apoptotic (Survival)	(179)
	Bcl-XL	↓	Anti-apoptotic (Survival)	(170)
	Cox2	↓	Enzyme (Inflammation; Survival)	(180)
	c-Myc	↓	Transcription Factor (Survival)	(181)
	HER-2/neu	↓	Receptor (Proliferation)	(182)
	CDKs	↓	Cell-cycle progression (Proliferation)	(173)
	VEGF	↓	Angiogenesis (Tumor growth)	(183)
	MMP9	↓	Angiogenesis and metastasis	(184)
	MMP2	↓	Angiogenesis and metastasis	
	bFGF	↓	Angiogenesis (Tumor growth)	
STAT2*	CD40	↑	TNF receptor (Apoptosis)	(150)
	CD80	↑	Ligand for CD28 (Apoptosis)	
STAT3	Mcl-1	↑	Anti-apoptosis (Survival)	(185)
	Bcl-2	↑	Anti-apoptosis (Survival)	(186)
	Bcl-XL	↑	Anti-apoptosis (Survival)	(187)
	Survivin	↑	Anti-apoptosis (Survival)	(188)
	Cyclin D1	↑	Cell-cycle progression (Proliferation)	(189)
	c-Myc	↑	Cell-cycle progression (Proliferation)	
	Pim1/2	↑	Cell-cycle progression (Proliferation)	
	P21	↑	Cell cycle arrest	(190)
	P27	↑	Cell cycle arrest	
	VEGF	↑	Angiogenesis (Tumor growth)	(191)
	bFGF	↑	Angiogenesis (Tumor growth)	(192)
	IL-17	↑	Angiogenesis (Tumor growth)	(193)
	IL-23	↑	Angiogenesis (Tumor growth)	(194)
	CXCL12	↑	Myeloid cell proliferation, survival	(195)
	MMP2	↑	Myeloid cell proliferation, survival	(189)
	Cox2	↑	Myeloid cell proliferation, survival	(196)
	HIF 1α	↑	Proliferation, angiogenesis	(188)
	IL-6	↑	Proliferation	(197)
	IL-10	↑	Anti-inflammatory Stimulation	(198)
	IL-21	↑	Proliferation, differentiation	(199)
	Notch1	↑	Proliferation, differentiation	(200)
	Rac1	↑	Cell Cycle Progression	(201)
	Socs1	↑	Pro-inflammatory	(202)
	Socs3	↑	Pro-inflammatory	
	P53	↓	Apoptosis Induction	(203)
	CD80	↓	Ligand for CD28 (Apoptosis)	(204)
	CXCL10	↓	Immuno-surveillance	(205)
	CCL5/RANTES	↓	Inflammatory Mediator	(206)
	CCL2/MCP1	↓	Inflammatory Mediator	(188)
	IFN gamma	↓	Immuno-regulatory, Anti-proliferation	(198)
	IFN betta	↓	Apoptosis Induction	(206)
	Fas	↓	Apoptosis Induction	(207)
	Fas-L	↓	Apoptosis Induction	
	BAX	↓	Apoptosis Induction	(208)
STAT4	IFN gamma	↑	Immuno-regulatory, Anti-proliferation	(209)
STAT5	Bcl-XL	↑	Anti-apoptosis (Survival)	(210)
	Bcl-2	↑	Anti-apoptosis (Survival)	(211), (212)
	Mcl-1	↑	Anti-apoptosis (Survival)	
	Survivin	↑	Anti-apoptosis (Survival)	
	Pim-1	↑	Cell-cycle progression (Proliferation)	
	c-Myc	↑	Cell-cycle progression (Proliferation)	
	Cyclin D1	↑	Cell-cycle progression (Proliferation)	(210)
	P21	↑	Cell-cycle progression (Proliferation)	(213)
	Id-1	↑	Cell growth, differentiation, survival	(214)
	Socs1	↑	Pro-inflammatory	(215)
	Socs3	↑	Pro-inflammatory	(216)
	OSM	↑	Pro-inflammatory	(217)
	P53	↓	Apoptosis Induction	(218)
STAT6	Bcl-2	↑	Anti-apoptosis (Survival)	(219)
	Bcl-XL	↑	Anti-apoptosis (Survival)	(220)
	GATA3	↑	Differentiation	(221)

BAX, Bcl-2-associated X protein; bFGF, basic fibroblast growth factor; Bcl-2, B-cell lymphoma 2; Bcl-XL, B-cell lymphoma extra-large; CD40 and 80, cluster of differentiation 40 and 80; CCL, chemokine ligand; CDKs, cyclin-dependent kinases; CDKN1, cyclin-dependent kinase inhibitor 1; Cox-2, cyclooxygenase 2; IFITM1, interferon-induced transmembrane protein 1; IFN, interferon; HER2/neu, human epidermal growth factor receptor 2; HIF1α, hypoxia-inducible factor 1-alpha; Id-1, inhibitor of DNA binding 1; IL, interleukin; IRF1, interferon regulatory factor 1; Mcl-1, Myeloid Cell Leukemia Sequence 1; MMP, matrix metallopeptidase; OSM, oncostatin M; Rac1, ras-related C3 botulinum toxin substrate 1, Socs, suppressor of cytokine signaling; TRAIL, TNF (tumor necrosis factor)-related apoptosis-inducing ligand; VEGF, vascular endothelial growth factor; XAF1, XIAP (X-linked inhibitor of apoptosis protein)-associated factor 1. *STAT2 does not induce transcription alone, and is incorporated into ISGF3—(STAT1/STAT2/IRF9) complex. Upward arrows indicate up-regulation, while downward arrows indicate down-regulation.

STAT2 was also initially identified as a component of the STAT1/STAT2 heterodimer and IFN-regulatory factor 9, and is the largest molecule among the proteins in this family. It has been reported that tyrosine phosphorylation and heterodimerization with STAT1 are necessary for STAT2 transportation into nucleus (167). However, non-phosphorylated STAT2 is also translocated into nucleus without interferon receptor signaling, as a result of interaction with a non-STAT transcription factor called IRF9 (222). There is little information available about the formation of STAT2 homodimers, and the role of STAT2 as an independent transcription factor is largely unknown and controversial (175). STAT2 activation has been linked to increased expression level of Cluster of differentiation (CD) 40 and CD80 (150), receptors involved in a multiple-step T-cell activation model (223). Similar to STAT1, impaired response to interferons observed subsequent to STAT2 knockdown in animal models has resulted in viral infections (224).

STAT3 is by far the most studied and best-known member of STAT family of proteins, and along with STAT5 have been extensively investigated in cancer biology. The outcome of STAT3 activation, however, is almost the exact opposite of STAT1 (despite almost 70% sequence homology, and similar crystal structure as tyrosine phosphorylated dimers) and contributes to carcinogenic processes and cancer progression (150, 225). It has been interconnected with nuclear factor-κB (NF-κB) signaling, and they seem to co-regulate a variety of oncogenic and inflammatory genes (226). It also seems to play a crucial role in development, as knocking down STAT3 in mice has been proven to be lethal to the embryo (227). STAT3 could be transported into nucleus both as tyrosine phosphorylated and non-phosphorylated, which is mediated by importin α3 (159) (silencing importin α3 using RNA interference approaches has shown to induce STAT3 accumulation in cytoplasm), while the main transporter for STAT1 is importin α5 (162). It has been reported that non-phosphorylated STAT3 present in nucleus could also affect the expression of many oncogenic proteins, independently, or after forming complexes with other transcription factors, e.g., JUN (228). Among well-known proteins that are overexpressed by STAT3 activation, Mcl-1, Bcl-2, Bcl-XL, and survivin are anti-apoptotic proteins that play a crucial role in cancer cell survival (186, 187, 189), cyclin D1 and c-Myc enhance proliferation (189), and VEGF promotes angiogenesis, which is required for tumor growth (229). On the other hand, STAT3 is reported to downregulate expression of important proteins involved in apoptosis induction or mechanism, including P53 (203), interferon β (206), Fas and its ligand, and BAX [(207, 208); Table 3).

STAT1/STAT2, STAT1/STAT3, (163), and STAT1/STAT4 (230) are the only heterodimers reported. Therefore, STAT4 is known to form homodimers, and the activation of this member of STAT family is triggered by IL-12 receptor, and is only linked directly to enhanced expression of interferon γ as a result (209), which is crucial in differentiation of T-helper cells 1 (231). On the other hand, STAT5 is the other member of the family usually associated with cancer. Two versions of this protein, known as STAT5A and 5B, are identified, which share a similar protein structure (more than 95% identical), and are reportedly involved in development and hematopoiesis, since impaired T-cell proliferation and severe anemia are reported in STAT5A/5B double-knockdown mice (232). STAT5B transport into nucleus is similar to STAT3, and can be transported in and out of nucleus in non-phosphorylated form as well (233). Also, similar to STAT3, STAT5 is also overactive in many invasive human cancers (163). STAT5 is involved in expression of many proteins that are linked to STAT3 as well, and therefore seems to contribute to similar outcomes (cell survival and enhanced proliferation). However, the expression of inhibitor of DNA binding 1 (Id-1) seems to be exclusively linked to activation of STAT5 (214). Id-1 is a protein involved in cancer progression, angiogenesis, and cell survival (234). STAT5 and STAT6 are both reportedly overactive in hematopoietic malignancies (226, 235). STAT6 activation seems to be triggered mainly by IL-4 and IL-13, and the loss of these cascades has been reported to impaired T-helper 2 cell differentiation (236) and development of certain types of leukemias and lymphomas (235), respectively. Majoros et al. have reviewed reports on “non-canonical” mechanisms of activation (including kinase-independent JAK functions and activity of non-phosphorylated STATs) recently (237).

MicroRNAs (miRNAs) are part of cellular gene expression regulators that can significantly change the phenotypic characteristics of the cell. They are expressed as hairpin structures, transformed in a multi-step process to a single strand RNA, and are incorporated into the RNA-induced silencing complex (RISC) to identify and bind to a partial or perfect complementary match on targeted mRNAs (238). Not only are miRNAs involved in the regulation of proteins involved in JAK/STAT pathway (similar to the majority of other cellular proteins), STAT family of proteins are also reported to regulate miRNA expression levels. For instance, it has been shown that STAT3 directly activates miRNA-21, which in turn, downregulate PTEN, among other proteins, which is a well-known tumor suppressor gene (239, 240). The interactions between STAT proteins and miRNAs have been previously reviewed (241).

## Targeting JAK/STAT pathway

Targeting members of JAK and STAT families of proteins with molecularly targeted drugs, and/or RNA interference (RNAi) approaches has been extensively studied, with many of them already in clinics or clinical trials. It has been hypothesized that blocking this signaling pathway could inhibit cancer progression as a single therapy, or in combination with other anticancer agents. The small molecule drugs targeting these proteins in clinical trials or used in clinics are summarized in Table 4. A quick look at the table reveals a few facts:

a. Members of JAK family have been a more popular target of molecularly targeted drugs than STATs. JAK is an upstream protein to STAT, which means it has to be activated in order to activate STATs, and this might be a hypothetical explanation for this exaggerated focus. However, STATs have been reported to be activated by other signaling mechanisms, independent of JAKs. The other explanation could be based on the hypothesis that upstream proteins might be involved in cross-talk with other signaling cascades, and therefore, by targeting JAKs we could also interfere with other mechanism involved in cancer progression. The emphasis on JAKs is also apparent in number of drugs in clinics and clinical trials compared to drugs targeting STATs (which are mostly still in pre-clinical stages);
b. While there is a variety of JAK proteins that have been targeted by small molecule drugs (including TYK2), the only member of STAT family that has been the focus of therapeutic attempts is STAT3 (with the exception of fludarabine that targets STAT1). This is mostly due to the fact that STAT3 has been one of most promising targets for molecularly targeted treatment. This also indicates less specificity seen in JAK inhibitors (especially pan-JAK inhibitors, e.g. ruxolitinib), rather than intentional targeting more than one JAK at a time;
c. JAK2/STAT3 seem to be the most popular targets in cancer treatment. In fact, cancer seems to be the dominant target for these therapeutic approaches. Autoimmune diseases (e.g., rheumatoid arthritis and psoriasis) are the second focus of attention.

**Table 4:** Selected small molecule drugs targeting JAK or STAT proteins used in clinical setting or in different stages of clinical trials.

	**Drug**	**Target protein**	**Stage**	**Application**	**Outcome**	**References**
JAKs	Ruxolitinib	JAK1/2	Clinic	P. Vera, Myelofibrosis	Effective with mild toxicity	(242)
	Tofacitinib	JAK3 > JAK1/2	Clinic	RA	–	(243)
			Phase III	Chronic Plaque Psoriasis	Efficient and Safe	(244)
	Fedratinib	JAK2	Phase III	Myelofibrosis	Reduced splenomegaly, encephalopathy (toxicity)	(245)
	Decernotinib	JAK1/2/3, TYK2	Phase II	RA	Improved symptoms	(246)
	Peficitinib	JAK1/2/3	Phase III	RA	Safe and efficient	(247)
	WHI-P154	JAK3	Mice	Glioblastoma multiforme	Delayed tumor progress	(248)
	CEP-33779	JAK2	Mice	Colorectal cancer	Suppressed tumor growth	(249)
	AG 490	JAK2	Mice	RA	Improved symptoms	(250)
			Rats	CLI	Enhanced blood flow	(251)
	WP1066	JAK2	Phase I	Brain tumors/melanoma	Underway (NCT01904123)	–
	Momelotinib	JAK1/2	Phase I/II	Myelofibrosis	Effective and tolerable	(252)
	Cerdulatinib	JAK1/2/3, TYK2	Phase I	CLL/B-cell NHL	Recruiting (NCT01994382)	–
	Filgotinib (GLPG0634)	JAK1 > JAK2/3, TYK2	Phase II	RA	Effective, well-tolerated	(253)
				CD	Clinical remission	(254)
	Pacritinib	JAK2	Phase III	Myelofibrosis	Terminated due to FDA concerns (NCT01773187)	–
	Baricitinib^*^	JAK1/2 > JAK3, TYK2	Phase III	RA	Improved symptoms	(255)
	Gandotinib (LY2784544)	JAK2	Phase II	Myeloproliferative Neoplasms	Ongoing (NCT01594723)	–
	TG101209	JAK2	Mice	Lung Cancer	Enhanced radiation effect	(256)
	XL019	JAK2 > JAK1/3, TYK2	Phase I	Myelofibrosis	Well-tolerated	(257)
	AT9283	JAK2/3	Phase II	Multiple Myeloma	No objective response	(258)
	AZ 960	JAK2	*In vitro*	Leukemia/Lymphoma	Growth arrest and apoptosis	(259)
	AZD1480	JAK1/2	Phase I	Solid Tumors	DLTs and lack of activity	(260)
	NVP-BSK805	JAK2 > JAK1/3, TYK2	Mice	P. Vera	Efficacious	(261)
	INCB018424	JAK1/2	Phase I/II	Myelofibrosis	Durable clinical benefits	(262)
	CEP-701	JAK2	Phase II	Myelofibrosis	Modest efficacy, but frequent GI toxicity	(263)
STATs	Fludarabine^†^	STAT1	Clinic	B-cell chronic lymphocytic leukemia	–	(264)
	S3I-201	STAT3 > STAT1/5	Mice	Breast cancer	Breast tumor regression	(265)
	STA-21	STAT3	Phase I/II	Psoriasis	Improvement of lesions with topical treatment	(266)
	OPB-51602	STAT3	Phase I	Hematologic and solid malignancies	Promising antitumor activity in NSCLS	(267)
	OPB-31121	STAT3	Phase I	Advanced solid tumors	Antitumor activity	(268)
	HO-3867	STAT3	Mice	Ovarian Cancer	Inhibition of tumor growth	(269)
	SH-4-54	STAT3 > STAT5	Mice	Glioma/Breast cancer	Inhibition of tumor growth	(270)
	SH5-07	STAT3	Mice	Glioma/Breast cancer	Inhibition of tumor growth	
	Niclosamide^‡^	STAT3	Mice	Head and neck cancer	Inhibition of tumor growth	(271)
	Cryptotanshinone	STAT3	Mice	Liver cancer	Effective STAT3 inhibition	(272)
	Stattic	STAT3	Mice	ESC Carcinoma	Radio-sensitization	(273)

*CD, Crohn's disease; CLI, critical limb ischemia; CLL, chronic lymphocytic leukemia; DLT, dose-limiting toxicity; ESC, esophageal squamous cell; GI, gastrointestinal; NHL, non-Hodgkin lymphoma; NSCLS, non-small-cell lung cancer; P. Vera, Polycythemia Vera; RA, rheumatoid arthritis*.

* *Baricitinib was approved for treatment of RA by European Commission in 2017*.

† *Fludarabine is a chemotherapeutic agent (purine analog) that primarily targets ribonucleotide reductase and inhibits DNA synthesis. However, an inhibitory effect on STAT1 has also been reported (274)*.

‡ *Niclosamide is a well-known anthelmintic agent (especially against tape worms) that has shown selective inhibition of STAT3*.

Ruxolitinib, tofacitinib, and fludarabine are the only molecularly targeted drugs against JAK/STAT pathway used in clinics. Fedratinib reached Phase III clinical trials; however, a report published in 2015 indicates that the clinical development has been discontinued due to toxic effects in some patients (most importantly encephalopathy), despite significant reduction of splenomegaly and symptom of myelofibrosis (245). Another interrupted development was recently reported for Pacritinib (a specific JAK2 inhibitor) in Phase III clinical trial, due to patient deaths, despite previous reports on its efficacy and safety in myelofibrosis (275). However, there are still four active Phase 1 and/or 2 trials that seem to continue on this drug. Peficitinib is another pan-JAK inhibitor in Phase III, which was recently reported efficacious in treatment moderate to severe rheumatoid arthritis (RA) with “acceptable safety profile” in a double-blind 12-wwek study in Japan (247). Recent reports also indicate development of TYK2-specific inhibitors, including NDI-031301 which has shown promising results in acute lymphoblastic leukemia (276). In addition to the small molecules included in Table 4, there are numerous new inhibitors of JAKs and STATs. A comprehensive review on investigational JAK inhibitors was recently published by Musumeci et al. (277).

An alternative approach in blocking signaling pathways involved in cancer progression is RNAi approaches that rely on temporary or permanent “silencing” of the targeted protein by targeting the mRNA responsible for the expression of the targeted protein. Due to a wider range of targets for these approaches, a larger number of effectors have been silenced via RNAi-based attempts, which include the downstream proteins activated by this pathway, and have been reviewed previously (278). Antisense oligonucleotides (ASOs) have also been studied for silencing proteins involved in this pathway. Recently, Hong et al. reported preclinical and initial clinical evaluation of methyl-modified ASOs (AZD9150) targeting STAT3 in patient-derived xenograft models and highly treatment-refractory lymphoma and non-small cell lung cancer patients (279). Another approach to this type of expression inhibition is known as “decoys.” Decoys targeting transcription factors, specifically, consist of nucleotide sequence derived from conserved regulatory elements, and block binding of transcription factor to genomic DNA by competitive inhibition. Sen et al. reported using cyclic decoys (by linking oligonucleotide strands using hexaethylene glycol spacers) in a “phase 0” study to target STAT3 in head and neck cancer patients (280). The newest strategy in silencing, the Clustered regularly interspaced short palindromic repeats (CRISPR) and CRISPR-associated protein (Cas)9 gene editing system, has been recently used to silence components of JAK/STAT pathway, mostly for investigational purposes. Quick et al. reported targeting JAK1 or STAT3 using CRISPR, which significantly reduced oncogene ubiquitin-specific protease 6 (USP6)/TRE17 in bone and soft tissue tumors, which indicates possibility of treatment of this type of malignancy by inhibition of JAK/STAT pathway (281).

## Role of JAKs in intracellular crosstalks

**Figure 2:**
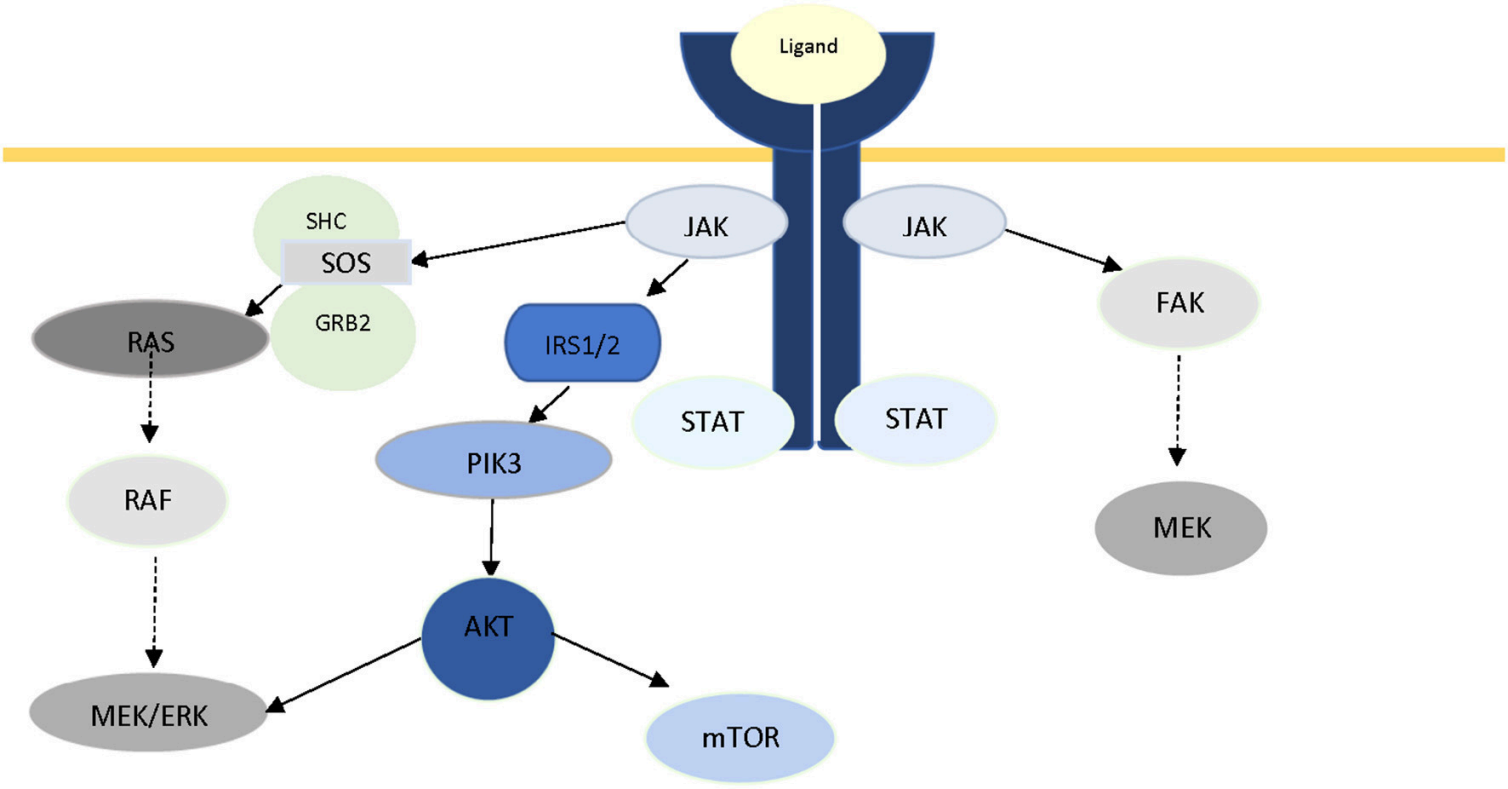
The role of JAK2 protein in intracellular crosstalk.

The pivotal role of JAKs in intracellular signaling is not limited in the JAK/STAT axis. Crosstalk between JAK and other well-known signaling pathways has been documented in recent years. In 2007, Levine et al. reviewed the role of JAK2 in myeloproliferative disorders, and reported activation of two other major signaling pathways (PI3K/Akt and Ras/Raf/MAPK/ERK) through JAK2 (282), which was later reported by Birzniece et al. (19) as part of growth factor signaling, and Chiba et al. in Alzheimer's disease (20). It has been suggested that JAK2-mediated ERK activation is conducted through Ras, and via SH2-domain containing transforming protein (SHC), growth factor receptor-bond protein (GRB), and son of sevenless (SOS) (283, 284). Activation of PI3K has been proposed to be via phosphorylation of IRS1/2 (285). In a review of IFN-mediated signaling, Platanias has reported the activation of the catalytic subunit (p110) of PI3K, and MAPKs via phosphorylation of VAV or other guanine-nucleotide-exchange factors (GEFs), as a result of activation of members of JAK family (286). Direct activation of FAK via JAK2 has also been reported in multiple studies (287–289). Figure 2 illustrates the central role of JAK protein in activation of these three major pathways. Additionally, there is ample evidence in literature for JAK-independent activation of STAT3 via Src (290, 291).

## Conclusion

JAK/STAT is a major and versatile signaling pathway that has been extensively studied in the past two decades for crucial roles in cancer and inflammation. The variety of the receptors triggering this pathway is unmatched among known signaling cascades, and the wide range of downstream proteins indicate the importance of JAK2/STAT3 axis in cancer progression. Despite promising tumor suppression in animal studies as a result of inhibition of this pathway, however, the safety issues have marred the success of this therapeutic approach in clinical settings to some extent. Also, due to the versatile nature of the pathway, and potential crosstalks with multiple alternative pathways, a monotherapy-based approach might not create reliable results on the long term. A more systematic exploration of intra- and inter-pathway connections would be helpful in understanding the molecular mechanisms of the signal transduction in this cascade, as well, as identification of novel targets in cancer therapy.

## Author contributions

Both authors made an intellectual contribution to this work. Literature search, tables and figures are mostly done by EB. The outline, the text and final editing was done by HM.

### Conflict of interest statement

The authors declare that the research was conducted in the absence of any commercial or financial relationships that could be construed as a potential conflict of interest.
